# Metastatic follicular thyroid cancer in a patient with Birt‐Hogg‐Dubé syndrome

**DOI:** 10.1002/ccr3.3454

**Published:** 2020-11-06

**Authors:** Elisa K. Bongetti, Rajesh Raj, Duncan R. Cooke, Mathew C. Mathew

**Affiliations:** ^1^ Launceston General Hospital Launceston Tas Australia

**Keywords:** Birt‐Hogg‐Dube syndrome, hereditary, kidney neoplasms, neoplastic syndromes, renal replacement therapy, thyroid neoplasms

## Abstract

Birt‐Hogg‐Dubé syndrome (BHDS) is an extremely rare genetic condition that predisposes to renal cell carcinoma. This case describes a novel case of a patient with BHDS who also develops follicular thyroid cancer.

## Graphical abstract

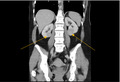

## 1 CASE HISTORY

A patient with Birt‐Hogg‐Dubé syndrome (BHDS) developed metastatic follicular thyroid cancer. BHDS is a rare hereditary cancer syndrome, which predisposes to renal cancer, pulmonary cysts, and cutaneous fibromas. This case provides a novel account of an unexpected malignancy in an extremely rare disease.

**FIGURE 1:**
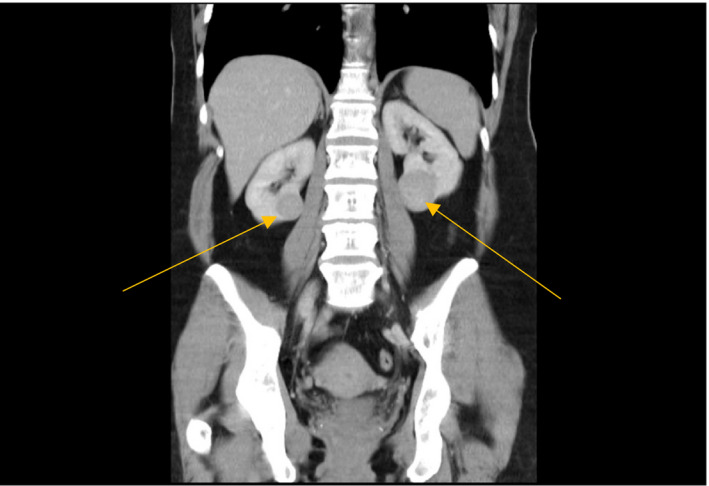
Computerized tomography scan of the abdomen and pelvis. Bilateral renal tumors are seen (arrows). Right tumor is 2 cm in diameter and left 3.6 cm diameter

A 45‐year‐old woman was detected to have bilateral renal masses on computed tomography (CT) (2 cm in diameter left and 3.6 cm in diameter right) when being investigated for lower abdominal pain (see Figure 1). The patient was previously well, with no significant past medical history and was not taking regular medications. There was no known family history of malignancy or kidney disease. Angiography demonstrated normal renal vasculature but peripheral enhancement of the renal masses. The patient underwent left partial nephrectomy, and a biopsy‐proven chromophobe renal cell carcinoma was excised. Three months later, she had a right partial nephrectomy. Intra‐operatively, it was discovered there were in fact two different lesions: first, a benign oncocytic tumor, and second, a hybrid oncocytic/chromophobe tumor (95% oncocytic, 5% chromophobe). Following her second partial nephrectomy, the patient suffered irretrievable kidney injury and was commenced on long‐term intermittent hemodialysis thrice a week.

The following year, the patient was referred for genetic counseling to consider whether the occurrence of bilateral renal tumors was secondary to a genetic disorder. During this consultation, it was noted on examination that she had multiple skin tags on her neck and chest. The patient was tested for the Birt‐Hogg‐Dube syndrome (BDHS) with genetic testing for the disease‐causing variant in the *folliculin (FLCN)* gene. Results from this testing were unable to confirm a diagnosis of BHDS. Nevertheless, it was recommended her sons receive screening with renal tract ultrasound from age thirty, every five years. The patient remained stable for two years, receiving thrice‐weekly hemodialysis at a satellite unit.

## 2 INVESTIGATIONS

The patient was subsequently considered for renal transplantation. Her case was reviewed by a team of specialist renal transplant physicians and surgeons, and it was recommended she undergo a nephrectomy to remove remaining renal tissue to minimize the risk of future malignancy. The surgery was complicated by splenic capsular tear, left adrenalectomy, and partial pancreatectomy. She suffered severe hypovolemic shock which required resuscitation in the intensive care unit but recovered and was later discharged following a prolonged admission to continue ongoing regular hemodialysis.

Additional investigations were undertaken to assess the patient for renal transplant. CT of the chest demonstrated bilateral pulmonary cysts. These cysts were asymptomatic and associated with normal lung function testing. Specialist respiratory and surgical opinions were sought, and no intervention was advised as the risk of pneumothorax was deemed low. Further genetic testing undertaken using multiplex ligation–dependent probe amplification testing in Germany revealed a deletion in major functional parts of *folliculin* gene in exon 14. Based on this information, a formal diagnosis of BHDS was made.

During an outpatient clinic appointment, a large left thyroid nodule was found on examination. Ultrasound‐guided fine‐needle aspiration of the mass demonstrated highly atypical epithelial proliferation, suspicious for thyroid follicular neoplasm. She had a total thyroidectomy and left neck dissection to remove a 60 mm, poorly differentiated follicular thyroid carcinoma. The patient was treated with liothyronine tablets, and an I^123^ scan did not demonstrate residual thyroid tissue in the neck.

## 3 OUTCOME AND FOLLOW‐UP

**FIGURE 2:**
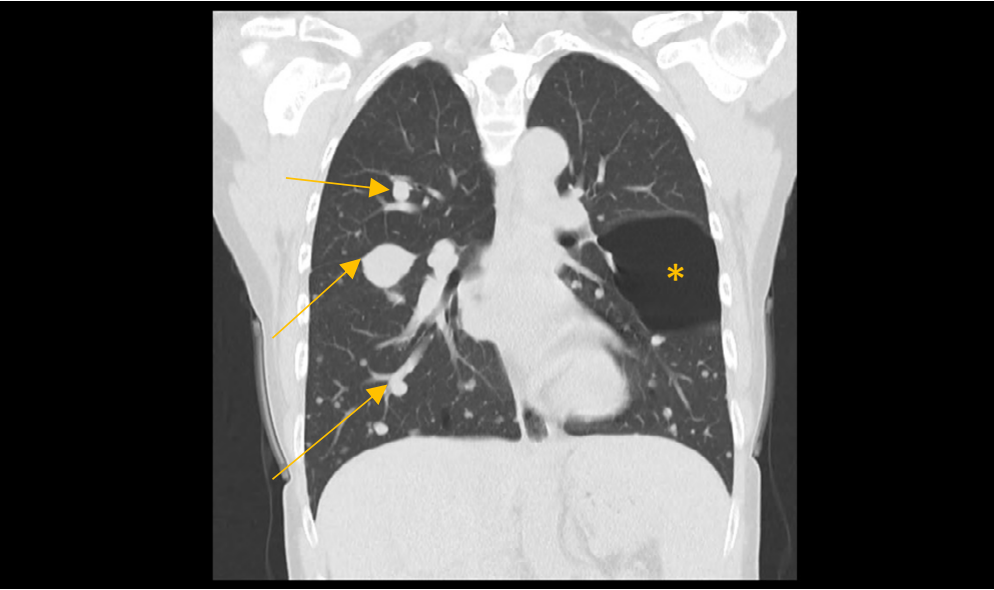
Multiple nodules are seen, throughout the lungs (arrows). The appearance is consistent with "showering" type metastasis, from thyroid primary cancer. At the midzone of the left lung, there is a large bulla (*), measuring approximately 6.24 cm, unchanged in appearance from previous imaging

Six months following her surgery, the patient reported severe back pain. CT showed multiple nodules throughout the lungs and lytic lesions in T8 and T12 vertebra (see Figure 2). Magnetic resonance imaging confirmed metastases to T1, T2, T9, and L1. Biopsy of a lung lesion confirmed metastatic follicular thyroid carcinoma. The patient received palliative lenvatinib, an oral multiple kinase inhibitor, and continued to live an active life. She survived for 15 months on this therapy while still receiving thrice‐weekly hemodialysis. She subsequently died from complications of her metastatic disease.

## 4 DISCUSSION

Birt‐Hogg‐Dubé syndrome an extremely rare autosomal dominant condition caused by a germline mutation in the tumor suppressor *folliculin (FLCN)* gene of which >150 disease‐causing variants have been discovered.^1^, ^2^, ^3^ BHDS predisposes to chromophobe renal cell carcinomas, benign oncocytic renal tumors, hybrid oncocytic renal tumors, pulmonary cysts, spontaneous pneumothorax, and cutaneous fibrofolliculomas.^4^ Some patients will only develop one or two of these manifestations, whereas others develop a full spectrum disease.^3^ Thirty percent of patients with BHDS develop renal cell carcinoma (RCC) with a mean onset of 46‐52 years of age.^4^ While thyroid nodules and cancers have been reported to occur in patients with BHDS, the syndrome is not believed to be causative of thyroid cancers.^4^, ^5^ This case highlights the unexpected finding of follicular thyroid cancer in a patient with BHDS and illustrates the challenges in managing a severe form of the hereditary cancer syndrome.

Renal cell carcinoma is uncommon and usually attributable to sporadic synchronous renal cell carcinoma, as only 2%‐5% of renal cell carcinomas are believed to be inherited.^1^ Up to five percent of patients with RCC have bilateral disease, and survival is similar to unilateral disease.^6^ Genetic syndromes known to cause RCC include von Hippel‐Lindau disease, tuberous sclerosis, hereditary papillary RCC, and BHDS.^7^ BHDS characteristically predisposes to multiple renal cancer histologies, as seen in the present case. While the detection of the *FLCN* gene mutation is diagnostic of BHDS, it is not essential as clinical criteria may be used.^3^ Genetic testing is not always reliable as false negatives may occur, as was illustrated in the present case.

There are reports of thyroid cancer in patients with BHDS ^2^, ^3^, ^4^, ^8^ In the family described by Birt and Hogg (1977), six of the nine family members with BHDS had medullary thyroid cancer.^2^ Moreover, Dong et al^3^ *(*2016) described two patients with BHDS who developed bilateral and multifocal papillary thyroid cancer. It was postulated that for patients with BHDS and thyroid cancer, total thyroidectomy with central neck dissection may be suitable as it is possible that there is a tendency for more extensive and severe thyroid disease.^3^ In the present case, a patient with BHDS developed metastatic follicular thyroid cancer, and although the malignancy was notably advanced by time of diagnosis, it is not possible to draw any conclusions about a causal association based on a single report. To our knowledge, this is the first reported case of follicular thyroid cancer with BHDS.

Patients with hereditary cancer syndromes who are being considered for solid organ transplantation require extensive screening and surveillance for malignancy to avoid devastating outcomes.^5^ In the present case, the patient was being considered for renal transplantation prior to her diagnosis of thyroid cancer. Currently, screening for thyroid cancer in BHDS is not included in recommendations for surveillance as there is no causal association between BHDS and thyroid cancer.^4^ Guidelines suggest abdominal imaging every 36 months for life utilizing computerized tomography or magnetic resonance imaging to monitor for renal cancers.^4^ Pulmonary assessment prior to any surgeries is also recommended due to risk of pulmonary cysts and spontaneous pneumothorax.^4^ Patients with von Hippel‐Lindau syndrome who underwent renal transplantation following bilateral nephrectomies had similar survival to age‐matched transplant recipients without the genetic condition.^9^ There have been no published outcomes of a patient with BHDS who has undergone renal transplants, so it is unknown what the outcomes for these patients are. Solid organ transplantation in patients with hereditary cancer syndromes is feasible, but as this case reinforces, an individualized approach to screening for other cancers is to be considered.

This case illustrates the complexity in managing a patient with a severe phenotypic presentation of BHDS. While there is no causal association that can be drawn on the basis of this case between thyroid cancer and BHDS, the case does support the notion proposed by Dong et al (2016) that aggressive treatment of thyroid cancer in this patient group may be considered. Moreover, for patients with BHDS being considered for renal transplantation, an awareness of reports of thyroid cancer in this patient group may prove invaluable.

## CONFLICTS OF INTEREST

No conflicts of interest to declare.

## AUTHOR CONTRIBUTIONS

EKB: oversaw the synthesis of the manuscript and review of relevant literature. RR: made significant contribution toward editing of the manuscript. DC: made significant contribution toward editing of the manuscript. MM: made significant contribution toward editing of the manuscript.
